# Heterosis in wheat: mechanisms, benefits, and challenges in hybrid development

**DOI:** 10.1093/jxb/eraf159

**Published:** 2025-04-15

**Authors:** Isobella Revell, Peng Zhang, Chongmei Dong, William T Salter, Richard Trethowan

**Affiliations:** IA Watson Grains Research Centre, Plant Breeding Institute, The University of Sydney, Narrabri, NSW 2390, Australia; The Plant Breeding Institute, School of Life and Environmental Sciences, The University of Sydney, Cobbitty Road, Cobbitty NSW 2570, Australia; The Plant Breeding Institute, School of Life and Environmental Sciences, The University of Sydney, Cobbitty Road, Cobbitty NSW 2570, Australia; IA Watson Grains Research Centre, Plant Breeding Institute, The University of Sydney, Narrabri, NSW 2390, Australia; School of Life and Environmental Sciences, Sydney Institute of Agriculture, The University of Sydney, Narrabri, NSW, Australia; IA Watson Grains Research Centre, Plant Breeding Institute, The University of Sydney, Narrabri, NSW 2390, Australia; The Plant Breeding Institute, School of Life and Environmental Sciences, The University of Sydney, Cobbitty Road, Cobbitty NSW 2570, Australia; Murdoch University, Australia

**Keywords:** Crop production, genetics, heterosis, hybrid breeding, hybrid vigour, hybrid wheat, *Triticum aestivum*

## Abstract

Heterosis, or hybrid vigour, has been exploited by plant breeders and grain growers for more than a century, and open-pollinated crops such as maize have been successfully commercialised. However, the full genetic potential of heterosis has yet to be realised and the fundamental mechanisms underlying this complex process are poorly understood. The challenges of hybrid seed production in self-pollinated crops, including cereals such as wheat and barley, have prevented the widespread exploitation of heterosis in these crops. Focussing on wheat, this review details current understanding of the genetic control of heterosis and explores the efficacy of different methods for producing F_1_ hybrids. We posit that the mechanisms underlying heterosis are probably a combination of multiple or all current theories, and that the conversion of inbred crops such as wheat into hybrid breeding systems can be further enhanced using a complete genic system, lessening the need for current, more complex hybrid production systems.

## Teaser

Heterosis is a complex biological phenomenon with unrealised potential for utilisation in large-scale crop production systems, and this review examines current knowledge in the context of wheat.

## Introduction

Hybrid crops are considered to be the most effective and efficient way to increase yields (Schnable and Springer, 2013). This is due to a complex, naturally occurring phenomenon known as heterosis, where the resulting first-cross offspring of two genetically distinct individuals (both within and between species) display an increase in vigour or in the value of a given measurable trait (East, 1936; Shull, 1948). Although it is unknown when heterosis was first observed, it is accepted that Charles Darwin’s published observations in the 19th century (Darwin, 1876) mark the beginning of scientific investigation and debate into the fundamental elements driving this phenomenon. Since its formal identification, many attempts to understand and explain heterosis have been published (Labroo *et al.*, 2021); however, a singular unifying theory is yet to be widely accepted (Hochholdinger and Baldauf, 2018; Labroo *et al.*, 2021; Wu *et al.*, 2021). Nevertheless, the exploitation and utilisation of heterosis and hybrid breeding in crop plants has been one of the most impactful innovations in modern agriculture (Hochholdinger and Baldauf, 2018).

Despite this, only a few large-scale agricultural crops and several horticultural crops have commercially successful F_1_ hybrid breeding programmes. Successful F_1_ hybrid breeding programmes in crops such as maize have revolutionised how plant breeders and researchers enhance important traits such as yield through exploitation of heterosis. The success of hybrid maize has driven the global effort to develop F_1_ hybrid breeding programmes in major self-pollinated cereal crops, including wheat and barley. One of the major hindrances to advances in wheat genetic gains via breeding for heterosis is the establishment of efficient and economically viable methods of hybrid seed production (Whitford *et al.*, 2013; Longin *et al.*, 2014). In the context of global wheat cultivation, hybrid crops only account for a minor proportion of the total grain produced. Given food insecurity in the world and the threat of a more variable and extreme climate, meeting the demand for consistent and stable yield gains in our most widely consumed crops is a significant challenge (Godfray *et al.*, 2010; Foley *et al.*, 2011; Ray *et al.*, 2012, 2013; Tadesse *et al.*, 2019). Hexaploid wheat (*Triticum aestivum*) provides 20% of global calories (Erenstein *et al.*, 2022) and enhancing genetic gains in this crop is therefore critical to reducing food insecurity (Tadesse *et al.*, 2019). Thus, an efficient wheat hybrid production system is needed to produce genetically superior and economically viable hybrid cultivars.

## Current wheat hybrid production systems

**Fig. 1.**
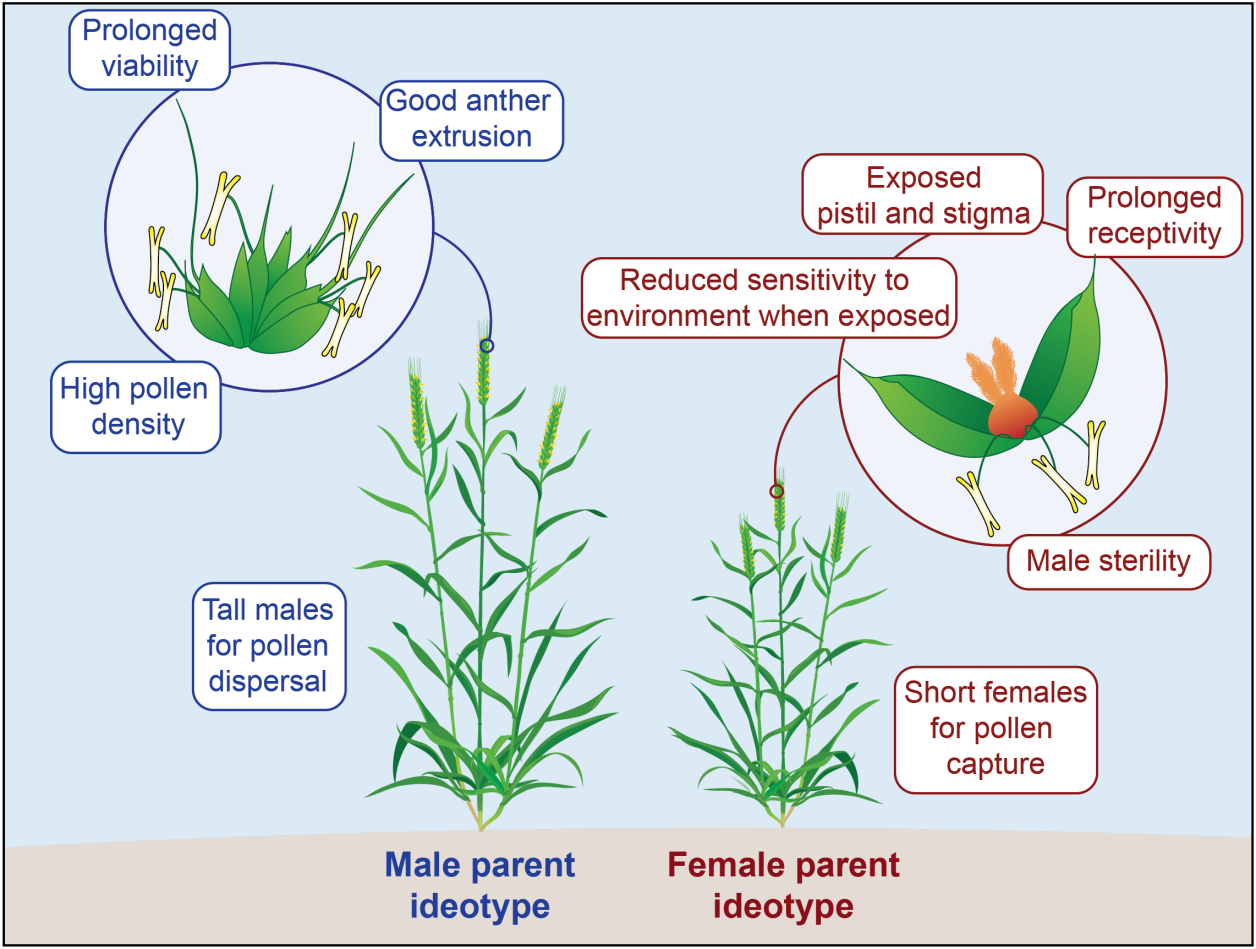
Male and female ideotypes for reproductive and physiological characteristics of parent wheat lines for successful hybrid seed production.

Wheat is an autogamous, self-pollinated, cereal crop species, which increases the complexity of producing F_1_ hybrids (Whitford *et al.*, 2013; Kitazaki *et al.*, 2023; Farooq *et al.*, 2024; Schneider *et al.*, 2024). Floral characteristics that promote outcrossing are advantageous to any commercial hybrid wheat breeding program (Whitford *et al.*, 2013; El Hanafi *et al.*, 2022; Garst *et al.*, 2023; Schneider *et al.*, 2024). In inbred wheat, fertilisation typically occurs before or at the time that the floret opens and anthers extrude (Whitford *et al.*, 2013; Langer *et al.*, 2014; Boeven *et al.*, 2016). Thus, accelerated and enhanced anther extrusion would be a desired male trait, whereas the female parent should ideally have gaping glumes that expose the stigma, combined with both extended stigma receptivity and anther longevity (Longin *et al.*, 2012; Whitford *et al.*, 2013; Langer *et al.*, 2014; Sade, 2019) (Fig. 1). In addition, having a female parent shorter than the male will enable gravity-assisted pollination. These male and female traits should optimise effective cross-pollination for hybrid seed production; however, the sensitivity of such floral components to the surrounding environment is a concern. There is a need for more research into floral characteristics (Whitford *et al.*, 2013; Schneider *et al.*, 2021, 2024) as optimisation of these traits could improve fertility in F_1_ seed production systems significantly.

**Fig. 2.**
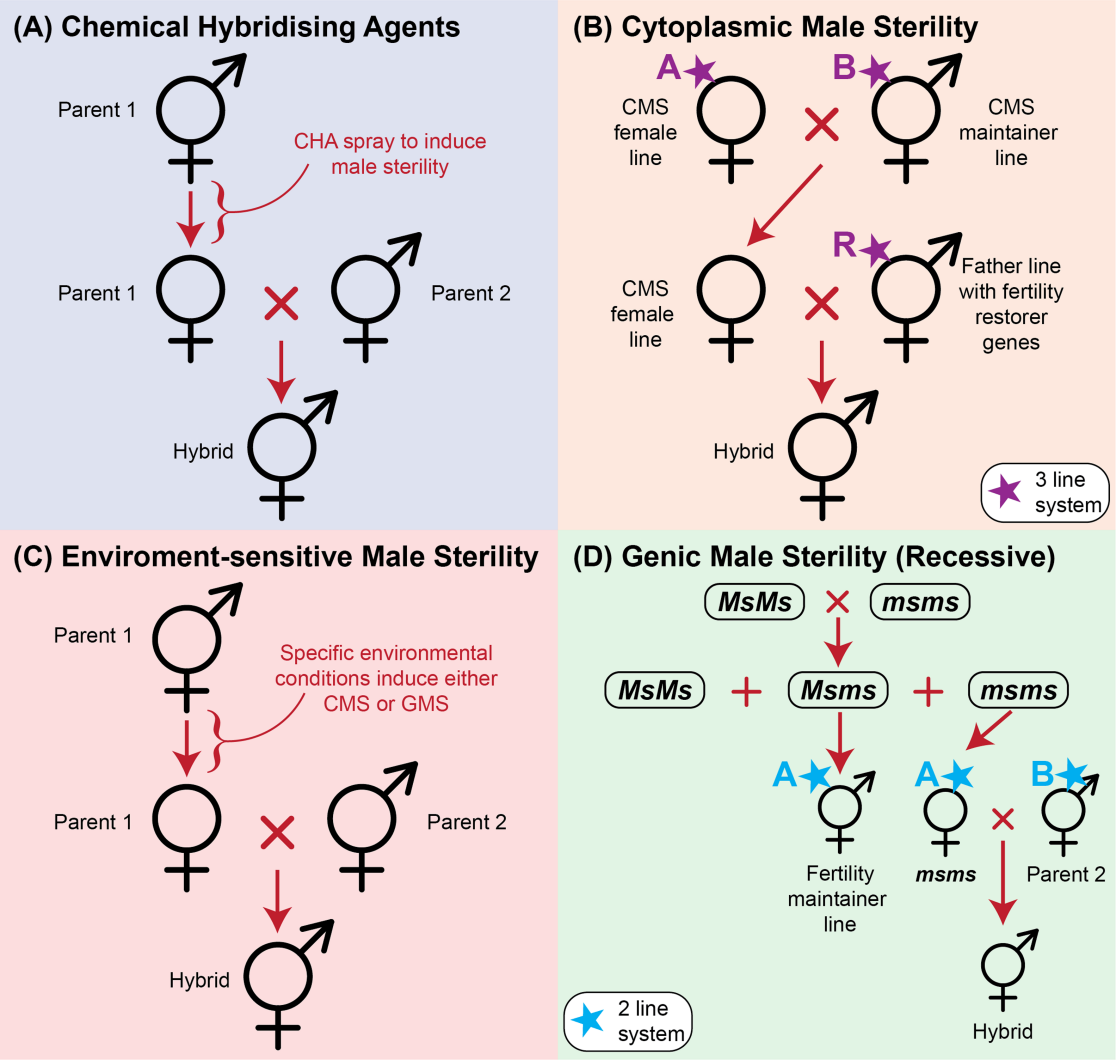
Four currently practiced approaches for producing hybrids in traditionally self-fertilised crops such as wheat. (A) Chemical hybridising agent (CHA) system, which induces male sterility in Parent 1 through chemical application. (B) Cytoplasmic male sterility (CMS) system, whereby mitochondrial DNA inducing the desired male-sterility is bred into the female parent and maintained using the A and B breeding lines. The resulting female is crossed with the male parent (R line), which must include fertility restorer genes. (C) Simplified overview of an environment-sensitive male-sterility approach. (D) Example of a genic male sterility (GMS) approach using recessive *ms* genes to induce male sterility in the female parent. In this system, the heterozygous offspring (*Msms*) of the cross to generate the female has fertility restoration genes enabling maintenance of the female line, and the homozygous recessive offspring (*msms*) is the female parent used for hybrid seed production.

The first step towards creating a hybrid production system in an autogamous crop species such as wheat is the development of a system to induce male sterility. The purpose of this is to inhibit self-fertilisation whilst simultaneously exposing the female gamete to cross-pollination with the elected male parent. There are a few currently practiced methods for producing and maintaining male sterility (Fig. 2); however, they all have shortcomings that hinder economically viable large-scale seed production, as discussed below.

### Chemical hybridising agents

Chemical hybridising agents (CHAs) are arguably the simplest form of inducing male sterility, and they comprise groups of chemicals that induce male sterility in the desired female inbred parent upon application by selectively affecting male gametes (Whitford *et al.*, 2013) (Fig. 2A). However, there are numerous variables that determine the efficacy of CHAs. It is important that: female gamete fertility is not affected; pollen sterility is complete; application rates and timing are optimised for different crop growth stages; the number of applications is minimised; there are no adverse effects on offspring seed quality and seedling or plant vigour; crop genotype-specificity requirements are reduced so that greater genetic diversity can be utilised; and no hazardous chemicals affect the surrounding environment (Gupta *et al.*, 2019; Sade and Doğan, 2024). There is yet to be a CHA that effectively meets all these requirements completely. As it stands, those that have been registered and utilised commercially are highly dependent on particular environmental conditions and genetic compositions to ensure their effective application and induction of male sterility (El Hanafi *et al.*, 2022), thus adding to the complexity and concerns regarding overall viability of CHAs as facilitators of male sterility. Currently, CHAs are the most widely utilised method of commercial hybrid wheat seed production, despite their limitations (Sade and Doğan, 2024).

### Cytoplasmic male sterility

Cytoplasmic male sterility (CMS) relies on naturally occurring chimeric genes found in mitochondrial DNA that induce varying levels of male sterility by causing defects in pollen production (Hanson and Bentolila, 2004; Whitford *et al.*, 2013; Melonek *et al.*, 2021). This requires maintaining three separate genetic lines to produce a fertile hybrid, the A, B, and R lines. Their purpose is to induce male sterility in the female parent (A line), maintain the female parent (B line), and restore male fertility in the resulting offspring (R line) (Fig. 2B). Whilst there are ~70 different cytoplasms known with this capability (Gupta *et al.*, 2019), many of them do not result in complete sterility in wheat (Whitford *et al.*, 2013). One that is commonly used effectively in a CMS system that does result in complete male sterility comes from *T. timopheevii* (Melonek *et al.*, 2021). Further complications include poor fertility restoration in F_1_ hybrids, the production of haploids, poor hybrid seed quality, and poor germination rates (Gupta *et al.*, 2019). In addition, the requirement of a female parent maintainer line in a fertile cytoplasm (B line) means that three separate breeding programmes are essentially required to produce hybrids. Despite this, successful commercialisation of CMS-based wheat hybrids has been reported, albeit they are relatively uncommon (Longin *et al.*, 2012; Melonek *et al.*, 2021).

### Environment-sensitive male sterility

There are three environment-sensitive male sterility systems that have been reported in the literature (Gupta *et al.*, 2019): photoperiod-sensitive CMS (PCMS), thermo-sensitive recessive genic male sterility (TGMS); and photoperiod and temperature-sensitive genic male sterility (PTGMS). These systems rely on strict environmental conditions to either induce cytoplasmic or genic male sterility (Fig. 2C), adding a level of complexity that is not required in CMS systems outlined above. Currently, these systems are used almost exclusively in China, with PTGMS reported as the only environment-sensitive male sterility system to have successfully produced a commercial hybrid (Gupta *et al.*, 2019). Despite these challenges, there have been successful breeding studies using one or more of these systems in rice, thereby further enhancing our understanding of heterosis (Mohanty *et al.*, 2025).

### Genic male sterility

Genic male sterility (GMS) is based on mutations in nuclear-encoded genes that have either occurred spontaneously or have been induced using physical or chemical mutagens (Whitford *et al.*, 2013) (Fig. 2D). Five mutants have been observed and retained in wheat populations, with three being dominant genes, *Ms2* (4DS), *Ms3* (5AS), and *Ms4* (4DS), and two being recessive, *ms1* (4BS) and *ms5* (3AL) (Whitford *et al.*, 2013; Gupta *et al.*, 2019). One advantage of GMS in comparison to other systems is that a much broader range of parental lines can be used, and thus it is more flexible and greater genetic variation can be included in the hybrid breeding program. GMS can confer both complete male sterility in the parental line and complete restoration of fertility in the F_1_ hybrid; however, issues still arise when maintaining the male-sterile parental line given that the male-sterile individuals need to be easily identified from the male-fertile segregants. There has been a breakthrough with regards to his issue, with a patent by Zhang *et al.* (2019) describing the use of a combination of genes for the BLue Aleurone (BLA) pigment with both male-sterility and fertility-restorer genes in one wheat plant. The BLA pigment is linked with the fertility restorer genes, thereby creating a visible marker to identify fertile grains (blue) from those that are male-sterile grins (white). Unlike the PTGMS and TGMS systems mentioned above, this GMS system is not limited by environmental restrictions. Given the fertility-restoration issues with CMS and chemical gametocides, an effective GMS system such as this remains the most probable avenue for producing commercially viable F_1_ hybrids in wheat.

## Understanding heterosis in wheat

Heterosis varies both between traits within species and between species for the same trait (Mackay *et al.*, 2021), and therefore it is better to consider the mechanisms of heterosis within a given species as opposed to generalisations across wider levels of taxonomy.

Traditional wheat breeding generates near-homozygous lines through multiple generations of self-fertilisation. This process has been known to cause inbreeding depression in some species, resulting in a loss of vigour and an increase in deleterious effects (Charlesworth and Willis, 2009). Consequently, traditional wheat breeding is a trade-off, where hybrid vigour is potentially lost to obtain desired alleles in a fixed homozygous state.

We know that defining heterotic groups, or pools, within a species can lead to considerable improvements in breeding for heterosis (John-Bejai *et al.*, 2024). Heterotic groups are sets of genotypes within a species that differ substantively from each other genetically. When crosses are made between two different heterotic groups, the resulting F_1_ hybrid is more likely to express heterosis. In essence, the divergence of germplasm into these distinct genetic groups increases their combining ability (Labroo *et al.*, 2021), and this can be both general and specific (Reif *et al.*, 2007). General combining ability (GCA) indicates that a parent produces a high frequency of superior progeny when crossed with a wide range of lines from another heterotic group. Specific combining ability (SCA), on the other hand, indicates that a particular parent combination produces heterotic progenies. Adding to this, if genetic distance increases, so does the probability of superior heterosis, although this is not guaranteed and not always the case (Zhao *et al.*, 2016; Boeven *et al.*, 2020; John-Bejai *et al.*, 2024). There has been a recent study showing the importance of deviating from relying solely on total genetic distance for parental selection and instead analysing in greater detail structural variations and the effect of the partitioned nature of the wheat genome on heterotic performance (Gimenez *et al.*, 2025). This study further adds to similar observations by Boeven *et al.* (2020) and John-Benjai *et al.* (2024) that there are major complexities surrounding the underlying mechanisms of heterosis that need to be examined in greater detail rather than simply calculating total genetic distance. However, it can still be posited that inbred parents with high GCA will more likely than not produce heterosis in wheat (Gowda *et al.*, 2012; Longin *et al.*, 2013).

**Fig. 3.**
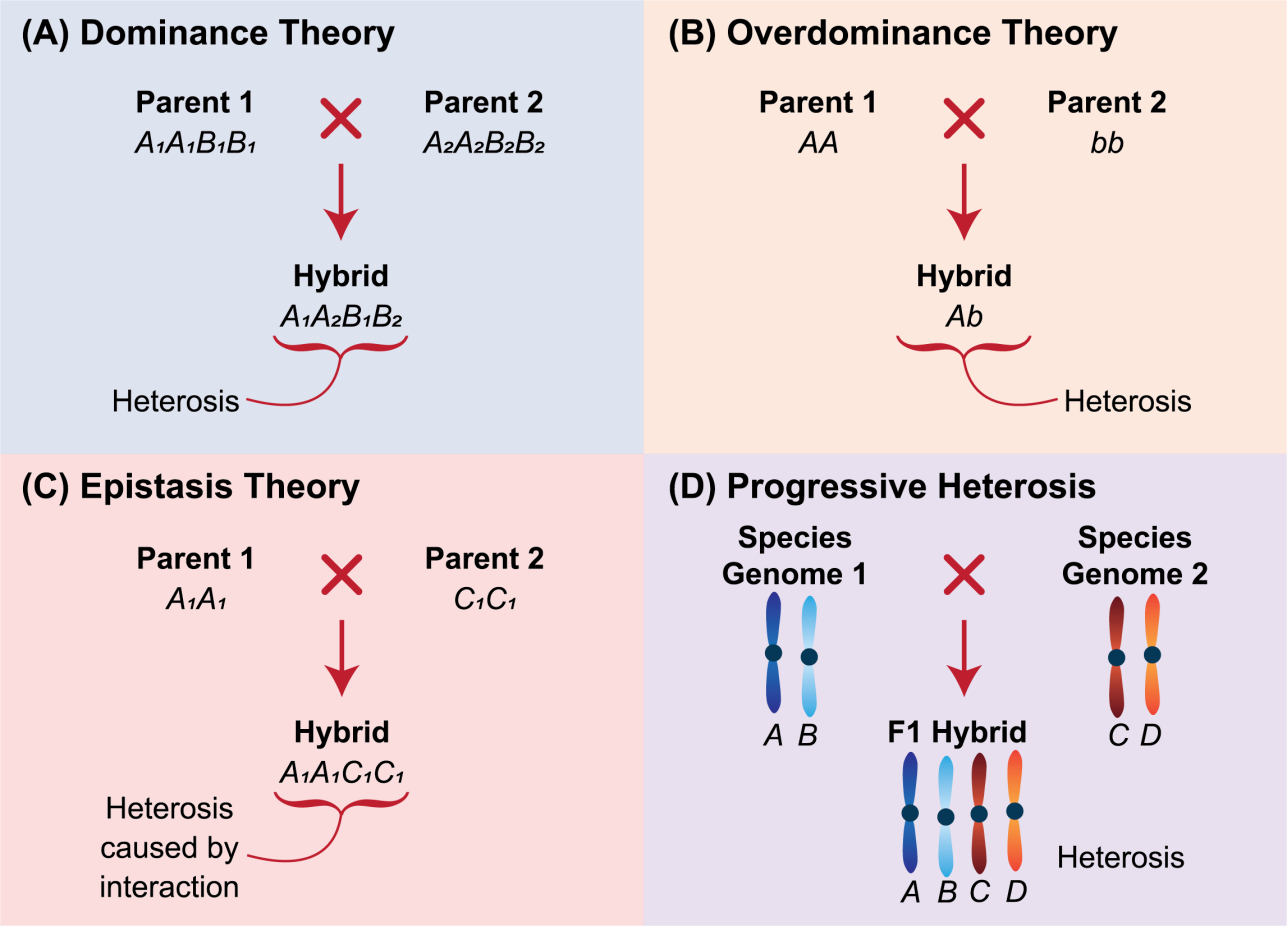
The four most common genetic theories for explaining the phenomenon of heterosis. (A) Dominance theory, in which a combination of multiple superior dominant alleles from both parents induces heterosis in the hybrid offspring. (B) Overdominance theory, in which Parent 1 is homozygous dominant, Parent 2 is homozygous recessive, and the resulting hybrid is heterozygous and expresses heterosis. (C) Epistasis theory, in which the genes in Parent 1 influence the effect of the genes in Parent 2 when combined in the hybrid offspring, and this interaction results in heterosis. (D) Progressive heterosis, in which polyploidisation results in heterosis in the hybrid.

There are two classical models proposed as explanations for heterosis, namely the dominance and overdominance models (Bruce, 1910; East, 1936; Shull, 1948), both of which were developed before advances in molecular biology. More recently, several genetic, molecular, and physiological explanations have been suggested as possible mechanisms underpinning heterosis (Fig. 3); however, to date, no single unifying theory has been accepted.

### The dominance model for heterosis

The dominance model hypothesises that superior dominant alleles hide the expression of less-fit recessive ones, so that only the former is expressed in the hybrid (Fig. 3A). Alternatively, in other cases, the hybrid contains excess loci expressing partial dominance in the same direction (directional dominance), leading to greater performance of progeny compared to parent (John-Bejai *et al.*, 2024). Thus, heterozygosity is not the driving force behind heterosis but instead the combination of multiple superior dominant alleles, or complementary homozygous recessive alleles, leads to the heterotic effect (Birchler *et al.*, 2010). Given the intensive selection, localisation, and subsequent inbreeding of cereals over centuries, we know that several highly dominant alleles have been lost over time and are no longer common in modern cultivars (Charlesworth and Willis, 2009). Under the dominance theory of heterosis, relative decreases in heterosis in F_1_ hybrids would then be expected (Charlesworth and Willis, 2009); however, this seems not to be the case in some crops (East, 1936; Birchler *et al.*, 2010). In maize, for example, inbred-line vigour has been continually improving over decades and yet levels of yield gains from heterosis in hybrid maize have remained constant (Duvick, 1999; Birchler *et al.*, 2010). Thus, whilst the dominance model certainly explains some of the observed heterosis, it does not tell the full story.

Furthermore, in attempting to apply the dominance theory to a polyploid crop such as wheat, more exceptions to the rule are observed. Birchler *et al.* (2010) reported a general trend of increasing heterosis with increasing genome number in allopolyploids, regardless of the frequencies of superior dominant alleles. The behaviour of heterosis in polyploids in many ways defies both the classical dominance and overdominance theories.

### The overdominance model for heterosis

The overdominance model hypothesises that alleles in a heterozygous state are superior to homozygosity, and that these loci interact to produce heterosis. There are examples of traits controlled by single genes where superior trait expression is observed in the heterozygous state compared to homozygosity (Mackay *et al.*, 2021) (Fig. 3B). This theory suggests that highly heterozygous wheat hybrids should consistently outperform homozygous or inbred parents; however, this is not the case and in fact it is actually a rare occurrence (Cowling *et al.*, 2020; Mackay *et al.*, 2021). What is probably happening in many cases is pseudo-overdominance, where a dominant allele effect is classified as overdominance because of linkage disequilibrium (Jones, 1917; Birchler *et al.*, 2010).

Much like dominance, the overdominance theory does not fully explain heterosis, and that remains the case even if both are considered together. Furthermore, both theories were first posited decades before the era of molecular biology and the availability of modern genetic tools.

### Epistasis and heterosis

Epistasis, or gene interaction, has long been considered important to understanding heterosis (Boeven *et al.*, 2020) (Fig. 3C); however, due to its complexity little is known of its influence. Nevertheless, a study has shown that epistatic effects could potentially have a larger influence on heterosis for yield in bread wheat than dominance effects (Jiang *et al.*, 2017). However, John-Bejai *et al.* (2024) were unable to conclude that epistatic interactions contributed to heterosis in their hybrid wheat population, instead finding additive genetic variance as a result of directional dominance to be the main contributor. This trend has also been observed in genetic studies of hybrid maize and hybrid rice, whereby incomplete dominance effects appear to be the major driver of heterosis (T. Lin et al., 2020; H. Liu et al., 2020). Given the contrasting results in these studies, it is perhaps not surprising that all conclude that further investigation into the role of epistasis in determining heterosis is required.

### Progressive heterosis

Progressive heterosis is the addition of diverse genomes through polyploidisation that result in higher levels of heterosis (Birchler *et al.*, 2010) (Fig. 3D). The evolution of wheat has combined three genetically distinct genomes across two hybridisation events. This process can therefore be classified as progressive heterosis (Briggle, 1963), or the creation of an ‘immortalised’ allopolyploid hybrid (Santantonio *et al.*, 2019). Historical studies observed this phenomenon when crossing different species of the same genus (interspecific crosses; East, 1936; Briggle, 1963). However, the heterosis observed is not necessarily yield-based and more with regards to growth rate and biomass production, thus further adding to the complexities surrounding explaining heterosis.

Interestingly, decline in vigour via self-pollination occurs at the same rate in polyploid species as it does in diploids, and hence inbreeding depression is not significantly different between different ploidy levels in traditionally autogamous crops (Birchler *et al.*, 2010).

### Effects of regulatory genes and gene balance on heterosis

The gene-balance hypothesis (Birchler and Veitia, 2010; Birchler *et al.*, 2010) has been proposed to highlight the potential effects of gene dosage on heterosis. The concept suggests that many heterotic genes exhibit partial dominance or recessive characteristics as opposed to complete dominance or recessive expression (J. Liu et al., 2020). The partial nature of trait expression suggests that alleles exhibit some form of dosage effect, which is highlighted in complex gene regulation networks (Birchler, 2016). It has been posited that a stoichiometric balance exists in regulatory gene networks and if this balance is disrupted through gene dosage then a change in gene expression is observed, and thus alterations to regulatory networks could indeed contribute to heterosis (Birchler and Veitia, 2007). This hypothesis explains some of gene functioning that underpins heterosis but does not explain all the variation that is observed. It does, however, add weight to the theory that heterosis is caused by multiple multigenic behaviours.

### Epigenetics and heterosis

Combining physiological and molecular analyses of heterosis is important to understanding its control in wheat and other species. Studies have indicated that hormone regulation has an impact on heterosis; for example, abscisic acid is a promoter of increased seedling germination rates in hybrid maize compared with the parental lines (Li *et al.*, 2016). Increased biomass, more-rapid cell division, and higher photosynthetic rates have all been observed to underpin vigour in Arabidopsis F_1_ hybrids (P-C. Liu *et al.*, 2020; Liu *et al.*, 2021). Liu *et al.* (2021) were able to demonstrate that biomass heterosis in Arabidopsis during early shoot development is related to a core difference in a transcriptional regulatory network between the hybrid and parents, despite an overall conservation of the transcriptional architecture that regulates early shoot development between them. Understanding the molecular basis of these physiological changes through RNA-sequencing and transcriptomic studies will further increase our knowledge of heterosis.

Continual advances in molecular biology are improving our understanding of heterosis. Evidence suggests that epigenetic factors such as DNA methylation, and histone and small RNA (sRNA) modifications play a role in regulating heterosis (J. Liu *et al.*, 2020; Rehman *et al.*, 2021; Wu *et al.*, 2021), with hybrids being shown to exhibit substantial differences in DNA methylation compared with their parents (Rehman *et al.*, 2021; Wu *et al.*, 2021). Increased knowledge of the regulation of gene expression in developmental pathways at both the transcriptional and proteomic levels could improve our understanding of heterosis (Knoch *et al.*, 2021; Rehman *et al.*, 2021; Wu *et al.*, 2021); however, once again this has not yet been achieved in wheat.

Although not established in wheat, studies of siRNA and sRNA in closely related species of tomato (Shivaprasad *et al.*, 2012) in maize (Barber *et al.*, 2012) have shown that their levels vary from parents to hybrid and that is correlated with hybrid performance. Interestingly, whilst the tomato study concluded that higher levels of sRNAs in the hybrid resulted in siRNAs also being more frequent, the maize study concluded that the hybrids expressing the greatest heterosis were actually down-regulating siRNAs in comparison to the parents.

Mitochondrial inheritance might also influence heterosis. An early study by Sarkissian and Srivastava (1967) showed that an optimal combination of mitochondrial architecture between two inbred parents induced a complementation phenomenon in a heterotic hybrid that increased oxygen uptake and phosphorylation. If the mitochondrial architecture of the two inbred parents was not complementary, then heterosis in the hybrid was not observed. Over a decade later, Srivastava (1981) was able to add to these findings by suggesting a similar concept to that of mitochondrial complementation but for chloroplasts.

A number of studies have also linked intrinsic biological processes such as circadian clock rhythms to heterosis. Thus, epigenetic regulation of circadian clock-mediated stress responses vary with genetic distance, causing heterosis (Miller *et al.*, 2015). This study focussed on Arabidopsis hybrids and concluded that heterotic hybrids were more efficient at balancing the trade-off between stress response and growth. Furthermore, a study of circadian-clock genes of rice hybrids showed that three had expression levels higher than those of the parents at the seedling stage in hybrids that expressed heterosis (Shen *et al.*, 2015).

Despite the many studies to date linking various epigenetic and molecular biological processes to heterosis, a clear understanding of the underlying genetic mechanisms driving these processes is still lacking and further investigations are needed.

### Identification of quantitative trait loci associated with heterosis

Much of the current research on heterosis is based on analysis of quantitative trait loci (QTLs). Treating heterosis as a complex quantitative trait has led to the identification of loci associated with both specific and general combining abilities in hybrids (Wu *et al.*, 2021). QTLs contributing to heterosis have been identified in hybrid rice, maize, cotton, tomato, and loquat (Krieger *et al.*, 2010; Sarfraz *et al.*, 2018; Wang *et al.*, 2018; Liu *et al.*, 2019; Z. Lin *et al.*, 2020); however, they are yet to be studied extensively in wheat because of the complexities of producing large numbers of hybrids. Thorwarth *et al.* (2019) have identified QTLs associated with grain protein content and yield using a hybrid wheat population to assist genomic selection in breeding for both these traits. However, this was not in the context of understanding heterosis but instead focussed on improving genomic selection methods by combining parental and hybrid data. To date, QTLs linked to heterosis in F_1_ hybrid wheat have not been reported.

Identification of QTLs that are associated with heterosis does provide a practical contribution to advances in hybrid breeding and could prove helpful in obtaining further evidence of the mechanisms driving heterosis, even though additivity is likely to be a minor factor in the heterotic response. Quantitative traits are incredibly complex and are influenced by environmental factors, genetic background, and ploidy (Wu *et al.*, 2021) and for these reasons they will only ever explain a small portion of the heterotic response.

## Conclusions

Genetic distance between parental inbred lines is generally a good place to start when breeding for heterosis (Boeven *et al.*, 2020); however, beyond that, there is no singular unifying theory that adequately breaks down all the components and mechanisms of heterosis. Moreover, several of the theories and contributing factors that we have outlined in this review are not necessarily consistent across all plant species, suggesting species-specific control of heterosis. Whilst molecular and physiological advances are helping our understanding of heterosis in many crop species, the relative lack of progress in wheat can, to some extent, be attributed to the difficulties of producing large numbers of hybrids and the complexity of its genome. Heterosis is a non-linear and largely quantitative trait that results from numerous effects that can be attributed to heterozygosity and gene interactions. A unified, all-encompassing, and species-specific approach that combines all the current theories concerning the drivers of heterosis is probably the most likely route towards a definite explanation of its control in wheat.
